# Supplementary material for: JOSA: Joint surface-based registration with atlas construction enables accurate alignment of the brain geometry and function
Source: ArXiv. 2023 Sep 12:arXiv:2303.01592v3. Preprint. [Version 3] (PMC10516111)
Supplement: Supplementary file 1 [file NIHPP2303.01592V3-supplement-1.pdf]

Supplementary Material

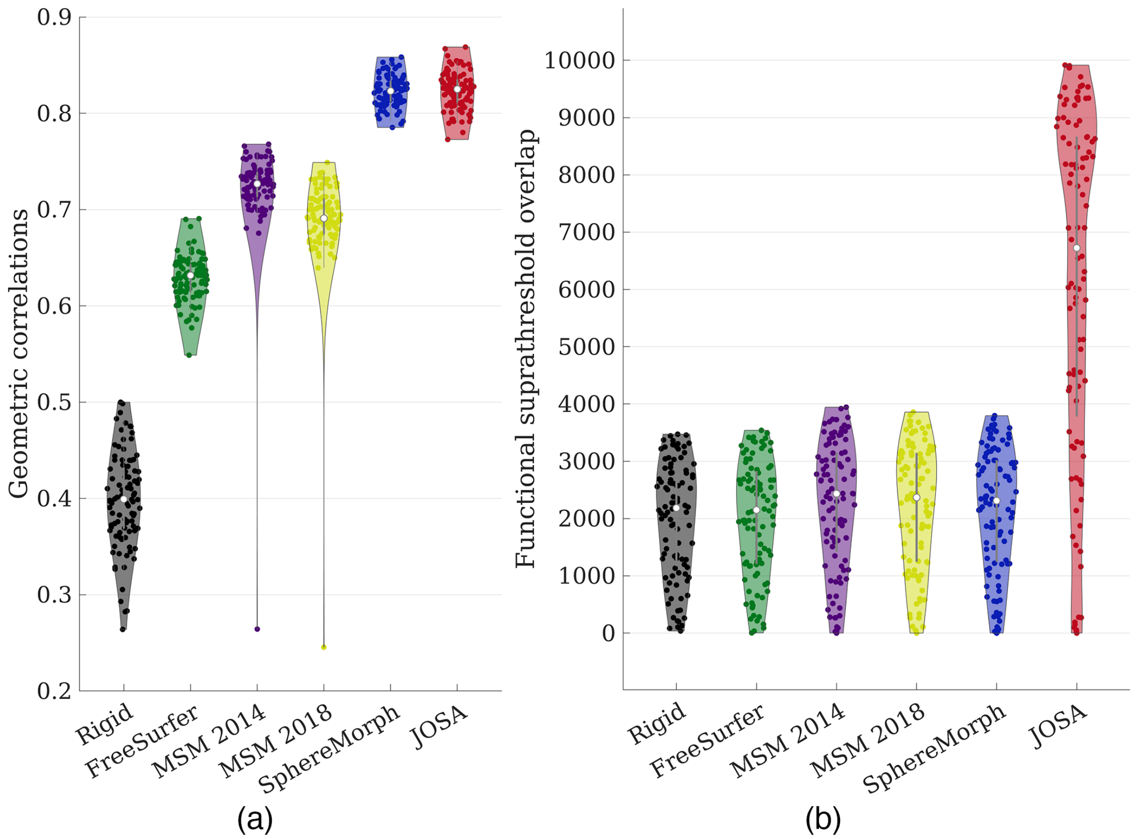

Figure S1: Violin plot of the original quantitative measures between individuals and group mean. (a) Geometric correlations; (b) Functional overlaps.

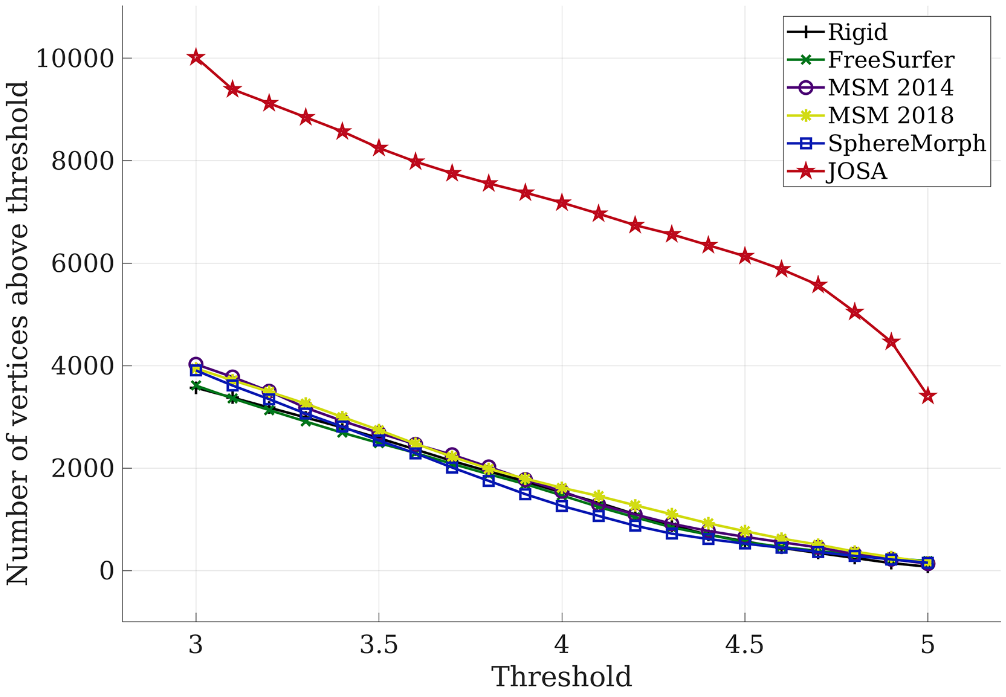

(a)

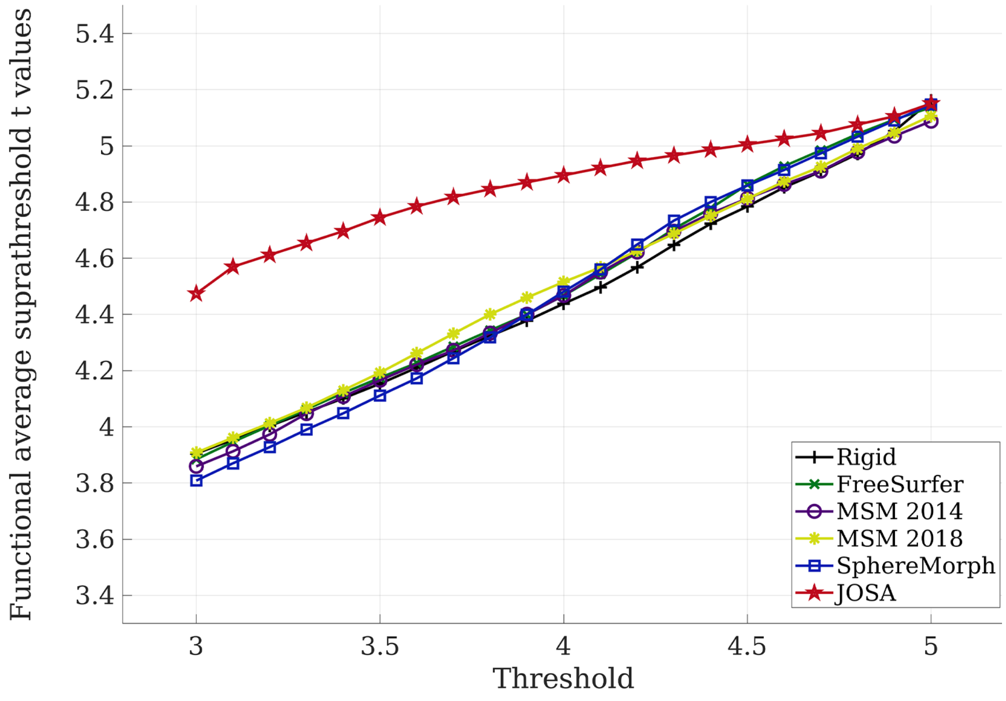

(b)

Figure S2: Impact factors of functional total suprathreshold t values as a function of the threshold. (a) Number of vertices within regions that have functional values above the threshold; (b) The averaged functional values within regions in (a).
